# Pregnancy in Women with Systemic Lupus Erythematosus: A Retrospective Study of 83 Pregnancies at a Single Centre

**DOI:** 10.3390/ijerph120809876

**Published:** 2015-08-19

**Authors:** Shanying Chen, Xuejuan Sun, Bide Wu, Xuejian Lian

**Affiliations:** Department of Nephrology, Zhangzhou Affiliated Hospital of Fujian Medical University, Zhangzhou 363000, China; E-Mails: dqydqy386@sina.com (S.C.); sxj0043@163.com (X.S.); wbd555@163.com (B.W.)

**Keywords:** pregnancy, systemic lupus erythematosus

## Abstract

*Objective*: To evaluate the outcome of 80 pregnant women with systemic lupus erythematosus (SLE) and explore the risk factors for lupus flare, obstetric complications and fetal loss. *Methods*: 83 pregnancies in 80 women were divided into three groups. Group A: patients in remission for > 6 months before pregnancy, proteinuria < 0.5 g per day, without renal failure and discontinuation of cytotoxic drugs for > one year; Group B: patients with SLE disease activity in the six months before pregnancy; Group C: patients with new onset SLE during pregnancy. *Results*: In group A, 76.47% pregnancies achieved full-term deliveries and 80.39% achieved live born infants. In group B and C, the outcome was poor. Among 62 patients (64 pregnancies) diagnosed as SLE before pregnancy, SLE flares occurred in 27 (42.19%) pregnancies. SLE disease activity in the six months before pregnancy was significantly associated with lupus flare (OR 5.00, 95% CI 1.14–21.87, *p =* 0.03) and fetal loss. New onset lupus during pregnancy was independently associated with obstetric complications (OR 7.22, 95% CI 2.14–24.38, *p =* 0.001). *Conclusions*: The current study confirmed the previous report that SLE should be considered a high risk of pregnancy. If pregnancy is planned after remission for > 6 months, the favorable outcome can be achieved.

## 1. Introduction

Systemic lupus erythematosus (SLE) is a common autoimmune disease and predominantly affects fertile women. The relationship of SLE with pregnancy is complex [1,2]. One concern is about the impact of SLE on pregnancy. Some previous studies indicated that pregnant women with SLE are at a higher risk of adverse pregnancy outcomes [3,4,5]. Pregnant women with SLE are high-risk patients, however, the outcome of pregnancy with SLE might be relatively good [6]. The other important concern is the impact of pregnancy on SLE. The Hopkins Lupus Pregnancy Centre’s experiences showed that lupus flare occurred in 60% of the pregnancies [7]. Other studies reported relatively lower flare rates and even unchanged condition during pregnancy [1,2,6].

It is difficult to compare previous studies because of highly heterogeneous study populations. The other potential explanation is the diversity of clinical manifestations of SLE and a variety of factors involved in the outcome. These factors include disease activity, lupus nephritis, antiphospholipid antibodies (aPLs), anti-Ro/SSA antibodies, and hypertension [1,2,8,9,10,11,12].

As we know, there is a paucity of data on pregnancy of Chinese women with SLE. The aim of this study was to evaluate the maternal and fetal outcomes of pregnant women with SLE. We also explored the risk factors for lupus flare, obstetric complications and fetal loss of pregnant women with SLE.

## 2. Patients and Methods

### 2.1. Patients

This was a retrospective study in a single centre. SLE was diagnosed using the 1997 revised American College of Rheumatology criteria [13]. We included pregnant women with SLE at the Zhangzhou Affiliated Hospital of Fujian Medical University between January of 2008 and December of 2013. Eighty three pregnancies in 80 women with SLE were included. Three women with SLE were pregnant twice. All data on age, personal history, blood pressure, laboratory variables, clinical manifestations and assessment of SLE activity were obtained from health records and discharge reports.

Before pregnancy, SLE activity was assessed using the SLE activity index (SLEDAI). SLE was considered as active if SLEDAI > 4 [14]. During pregnancy, the assessment of lupus flare was based on routine monitor [15]. We also use assessed multi-system manifestations including psychiatric or central nervous system, skin or mucous lesion and polyserositis. In the current study, lupus flare was considered if one of the following was present: (1) new onset proteinuria or /and hematuria; (2) psychiatric or central nervous system manifestations (not caused by pre-eclampsia, eclampsia, or HELLP sydrome); (3) Leukocytopenia or thrombocytopenia or Coomb’s positive hemolytic anemia; (4) New onset skin or mucous lesion; (5) Polyserositis; (6) Fever > 38.0 °C (not caused by infection or drug). Based on the disease status, all patients were divided into three groups. Group A: patients in remission for more than 6 months before pregnancy, proteinuria < 0.5 g per day, without renal failure and discontinuation of cytotoxic drugs for more than one year; Group B: patients with SLE disease activity in the six months before pregnancy; Group C: patients with new onset SLE during pregnancy. 49 patients with mean age of 28 years were included in group A, 13 patients with mean age of 26 years were included in group B, and 19 patients with mean age of 27 years were included in group C. Three women with SLE had two times of pregnancy. Among these six pregnancies, five of them were included in group A, and one of them was included in group C.

### 2.2. Assessment of SLE

Assessment measures of organ damage in SLE included dermatological symptoms, musculoskeletal damage, hematological damage, kidney and nervous system damage. Lupus nephritis (LN) was defined as abnormal urinalysis and/or proteinuria and/or renal failure. Proteinuria was defined as *>* 0.5 g of *protein/day* or *urine dipstick analysis indicated 1+–3+ for protein. Nephrotic syndrome (NS) was diagnosed if two of the following were present: (1) nephrotic range proteinuria (*proteinuria *> 3.5 g/24 h); (2) Serum albumin < 30 g/L*. Renal failure was defined as serum creatitine>1.5 mg/dL. The following laboratory variables were included: completed blood count, urinalysis, serum albumin, erythrocyte sedimentation rate (ESR), C-reactive protein (CRP), serum creatitine, urinary protein, ANA, anti-double-stranded DNA body, anti-Ro/SSA antibody, aPLs, anti-Smith antibody and complement C3. Hypertension was defined as a systolic blood pressure ≥140 mm Hg and/or a diastolic blood pressure ≥90 mm Hg or as a self-reported history of hypertension [16]. Gestational hypertension was defined as new onset hypertension during pregnancy. Leukocytopenia was defines as leucocyte count < 4 × 10^9^/L. Thrombocytopenia was defined as platelet count < 10 × 10^9^/L. All drugs administered pre-conception, during pregnancy and puerperium were recorded.

### 2.3. Maternal and Fetal Outcome

Maternal outcomes included mortality, lupus flare, and obstetric complications (gestational hypertension, pre-eclampsia, eclampsia, and HELLP syndrome). The first trimester was defined from conception until the 13th week; second trimester was referred to conception from the 14th until the 28th week; third trimester was conception from the 28th week until delivery; puerperium was defined as 6 weeks post-delivery or post-abortion [17].

Fetal outcomes included therapeutic abortion, elective abortion, spontaneous abortion, prematurity, stillbirth, and small-for-date infant. Therapeutic abortion was defined as the termination of a pregnancy by medical consultation. Elective abortion was referred to the termination of a pregnancy requested by patients. Spontaneous abortion was defined as the natural death of a fetus before the end for 28th weeks of gestation and weight of fetus was <1000 g. Prematurity was defined as the birth of a baby between the 28th and 37th week of gestation [18]. Stillbirth was defined when a fetus died in the uterus after 28th week of gestation. Small-for-date infant was referred to a baby that was born after 37th week of gestation and weight was <2500 g.

### 2.4. Follow-Up

We reviewed outpatients’ records and laboratory data as our follow-up data. Some of patients received telephone follow-up. All patients were followed up to December 2013.

### 2.5. Statistical Analysis

Data analyses were performed by using Stata (version 11.0). A two-tailed *p* value < 0.05 was considered significant. Baseline characteristics of patients were examined in three groups (group A, B and C). The Kruskal-Wallis Rank-Sum test was used for age and Fisher’s exact test was used for categorical variables. The differences in maternal and fetal outcomes among three groups were also examined. The chi-squared test or Fisher’s exact test was used. Fetal outcome of patients with new onset SLE during pregnancy was also listed and analyzed. Fisher’s exact test was used to compared to the differences in fetal outcomes among patients with new onset SLE during pregnancy. To examine the risk factors associated with lupus flare, obstetric complications and fetal loss for pregnant patients with SLE, logistic regression models were used. Firstly, variables were examined in unadjusted model. These variables included age, lupus nephritis, new onset SLE during pregnancy, and remission for less than six months before pregnancy. The presence of lupus flare, obstetric complications (including gestational hypertension, pre-eclampsia, eclampsia, and HELLP syndrome) and fetal loss were used as an independent variable, respectively. Next variables were examined in the adjusted models. In the study population, no woman was a smoker or a drinker and no patient had history of diabetes or gestational diabetes, so lifestyle (current smoker and alcohol use) and diabetes were not included in the models. There were high rates of therapeutic abortions in these groups and this might lead to erroroneous results, so we also ran models to explore the risk factors of fetal loss in patients without ending in therapeutic/elective abortion.

## 3. Results

### 3.1. Patient Characteristics

During the study period, 51 pregnancies in 49 patients were included in group A, 13 pregnancies in 13 patients were included in group B, and 19 patients were included in group C (Table 1). The difference in age among three groups was not significant.

**Table 1:** Baseline characteristics of pregnant women with SLE before pregnancy.

	Group A	Group B	Group C
Patients (Pregnancies)	N = 49 (51)	N = 13 (13)	N = 19 (19)
Age (years) *	28.31 ± 3.61	25.85 ± 5.11	26.90 ± 3.00
SLE duration (years)	5 (4–9)	2 (2–7)	0
Smoker	0	0	0
Hypertension ( before pregnancy)	0	2	0
Proteinuria	0	2	0
Renal failure	0	0	0
Drugs taken from 6 months before pregnancy to onset of pregnancy			
Prednisone/methylprednisolone	30	11	0
Hydroxychloroquine	7	0	0
Methotrexate	0	3	0
Mycophenolate mofetil	0	1	0
Leflunomide	0	1	0

* *p =* 0.12.

In group A, 48 pregnancies were in remission for more than 12 months before pregnancy. No patients had proteinuria or/and renal failure before pregnancy. No patients had symptoms of SLE. Before pregnancy, all patients had discontinued the cytotoxic drugs for more than one year and 19 patients with 21 pregnancies had discontinued drugs for SLE. Twenty three patients received low-dose glucocorticoid monotherapy (prednisone 10 mg qod-qd, or methylprednisolone 4–8 mg qd). Seven patients received a combination of glucocorticoid and hydroxychloroquine. The dose of hydroxychloroquine was 0.2 qd-bid. Before pregnancy, no patients had hypertension. No patients took aspirin and azathiprine.

In group B, six patients received glucocorticoid monotherapy (prednisone 10–25 mg qd); three patients received combinations of glucocorticoid and methotrexate (MTX); one patient received a combination of glucocorticoid and mycophenolate mofetil (MMF); one patient received a combination of glucocorticoid and leflunomide. Only two patients discontinued drugs for SLE, two patients had proteinuria and one patient had dental ulcers, skin lesions and edema. No patients took aspirin and azathiprine. Two patients had hypertension before pregnancy, and one of them took benazapril. One patient received renal biopsy two years before pregnancy and was diagnosed lupus nephritis IV (crescentic glomerulonephritis). In group C, no patients had history of hypertension and kidney diseases before pregnancy.

### 3.2. The Impact of SLE on Pregnancy

#### 3.2.1. Fetal Outcome (Table 2)

In group A, 76.47% (39/51) pregnancies achieved full-term deliveries and 80.39% (41/51) pregnancies achieved live born infants (including one bigeminal pregnancy). Five neonates were small-to-date infants. In group B, only 23.08% (3/13) pregnancies achieved full-term deliveries and 30.77% (4/13) pregnancies achieved live born infants.

**Table 2:** Fetal outcome of the 83 pregnancies.

	Group A	Group B	Group C	*p* Value
Pregnancies/Fetus	*N* = 51/52	*N* = 13/13	*N* = 19/19	
Full-term deliveries (vaginal delivery/caesarean)	39 (21/18)	3 (1/2)	6 (3/3)	<0.001
Small-to-date infant	5	1	1	
Prematurity	4 (5 neonates)	2	5	0.10
Live born infants	42	4	9	0.17
Still birth	2	1	2	0.43
Therapeutic abortion	4	5	6	0.005
Elective abortion	2	0	0	1.00
Spontaneous abortion	1	2	0	0.17
Loss-to-follow up	1	1	2	0.23

In group C, 31.58% (6/19) pregnancies achieved full-term deliveries and 47.37% (9/19) pregnancies achieved live born infants. Two women were lost to follow-up after discharge. One patient with antiphospholipid syndrome had a stillbirth and the other patient with antiphospholipid syndrome refused abortion and was lost to follow-up.

In the three groups, no neonate had malformation and neonatal lupus (including complete heart block). Patients in group A had the highest fetal survival rate.

#### 3.2.2. Maternal Outcome and Obstetric Complications (Table 3)

No death case was reported. Nine (17.65%) pregnancies in group A, three (23.08%) patients in group B and 12 (63.16%) patients in group C had gestational hypertension. Five (9.80%) pregnancies in group A, two (15.38%) patients in group B and six (31.58%) patients in group C had pre-eclampsia or eclampsia and only one patient in group C was diagnosed HELLP syndrome. Group C had the highest incidence of obstetric complications.

**Table 3:** Clinical manifestations during pregnancy.

	Group 1	Group 2	Group 3	*p* Value
Patients (Pregnancies)	*N* = 49 (51)	*N* = 13 (13)	*N* = 19 (19)	
**Obstetric complicates**				
New onset hypertension	9	3	12	0.001
Pre-eclampsia	4	2	5	0.18
Eclampsia	1	0	1	0.63
HELLP syndrome	0	0	1	
**Lupus manifestation**				
Leukocytopenia	1	1	6	0.006
Thrombocytopenia	6	3	7	0.18
Hematuria	13	8	17	0.01
Proteinuria	18	11	18	0.04
Nephrotic syndrome	10	7	14	0.02
Renal failure	3	1	1	0.96
Heart failure	2	0	4	0.054
Polyserositis	3	2	6	0.06
New onset skin lesions	3	4	9	0.004
Arthralgia	0	2	4	0.005
Dental ulcer	0	1	1	0.20
Hepatitis	0	0	1	0.20
**Laboratory**				
Anti-dsDNA	9	6	11	0.052
Anti-Smith	5	5	8	0.03
Anti-SSA	-	-	12	
Anti-SSB	-	-	3	
Anti-phospholipid antibody	-	-	2	
Hypocomplementamia				
C 3			16	
C4			15	

In group A, two patients had two times of pregnancies during the study period. Neither of them had obstetric complications. Both of them had one full-term pregnancy with a live born infant. One patient had an elective abortion and the other patient had a spontaneous abortion.

### 3.3. The Impact of Pregnancy on SLE (Lupus Flare)

Among 62 patients (64 pregnancies) diagnosed as SLE before pregnancy, SLE flares occurred in 27 (42.19%) pregnancies. During pregnancy, 17 (33.33%) pregnancies in group A had lupus flare*.* The clinical manifestations included proteinuria, hematuria, polyserositis, leukocytopenia, thrombocytopenia and new onset skin lesion. One patient only had new onset skin. Two patients having two pregnancies during the study period had no lupus flare. Three patients with NS developed renal failure during pregnancies. Nine patients had gestational hypertension.

In group B, lupus flare occurred in 10 (76.92%) patients and 11 patients had LN. In one patient, new onset skin lesion was the only manifestation of lupus flare. One patient with NS had renal failure.

In group C, 18 patients had proteinuria and 14 of them were diagnosed as NS. Twelve patients had new onset hypertension. Twelve patients had anti-Ro/SSA antibody, three patients had anti-SSB antibody and two patients had aPLs. In one patient, multi-systems were involved in (hepatitis, severe thrombocytopenia, nephrotic syndrome, inferior wall acute myocardial infarction).

### 3.4. Therapy Protocol (Table 4)

Pre-conception therapy was maintained as much as possible. Glucocorticoid and hydroxychloroquine were unchanged during pregnancy in all but six patients. Four patients in group A discontinued glucocorticoids because of fear of side effects and none of them developed lupus flare. In group B, two patients refused to continue taking glucocorticoids and one of them had lupus flare during pregnancy (NS and thrombocytopenia). Methotrexate, mycophenolate mofetil and leflunomide were discontinued. No prophylactic glucocorticoids were administered.

**Table 4:** Therapy of SLE patients during pregnancy and postpartum.

	Group 1	Group 2	Group 3
Patients (Pregnancies)	*N* = 49 (51)	*N* = 13 (13)	*N* = 19 (19)
**During pregnancy**			
Prednisone (<0.5 mg/kg)	21	4	0
Prednisone (1 mg/kg)	8	7	16
High-dose intravenous methylprednisolone	2	2	1
Anti-hypertensive agents	3	3	11
Low molecular weight heparin	0	2	1
Hydroxychloroquine	2	2	7
Hemodialysis	0	0	1
Plasmapheresis	0	1	0
**Postpartum**			
Prednisone/prednisone	32	13	17
Cyclophosphamide	1	2	0
Methotrexate	1	1	0
Azathioprine	0	0	1

SLE flares were treated with glucocorticoids. Two patients with NS (group A) and two patients (group B) with thrombocytopenia received high-dose intravenous methylprednisolone therapy (80–500 mg/day). One patient with NS (group B) received plasmapheresis four times. One patient began to be treated with glucocorticoids immediately after abortion and the doses of glucocorticoid were increased in two patients after delivery.

Seventeen patients of group C received glucocorticoids during pregnancy. Two patients were not received any treatment for SLE due to missed diagnosis. Their conditions improved post-delivery. Six months later, one patient returned to hospital with complaints of edema. The other patient saw a doctor because of facial erythema after one year. SLE was confirmed after examination.

### 3.5. New Onset SLE during Pregnancy (Table 5)

Although patients with new onset SLE during pregnancy had significant lower rate of live born infant than patients in group A. In eight patients with new onset SLE during the third trimester of pregnancy, 87.5% (7/8) patients achieved a live born infant.

**Table 5:** Fetal outcome of patients with new onset SLE during pregnancy.

	First	Second	Third	*p* Value
Pregnancies/Fetus	*N* = 3/3	*N* = 8/8	*N* = 8/8	
Full-term deliveries	0	1	5	0.04
Small-to-date infant	0	0	1	1.00
Prematurity	1	1	3	0.50
Live born infants	1	1	7	0.006
Still birth	0	1	1	1.00
Therapeutic abortion	2	4	0	0.04
Loss-to-follow up	0	2	0	-

### 3.6. Follow-Up Post Delivery

In group A, during post-delivery follow up, five patients still had proteinuria and urine dipstick analysis indicated 1+−3+ for protein.

In group B, urine protein test was negative in six patients and two patients lost to follow-up. Urine dipstick analysis indicated 1+−3+ for protein in five patients. Among these five patients, one patient had onset fever and proteinuria one year later delivery and received combination of glucocorticoid and cyclophosphamide therapy. The patient with crescentic glomerulonephritis developed to chronic renal failure three years later delivery.

In group C*,* urine protein test was negative in seven patients during follow-up and seven patients still had proteinuria. One severe patient with involved multi-systems improved after treatment and urine dipstick analysis showed 1+ for protein. One patient received renal biopsy and was diagnosed lupus nephritis IV + V. One patient died because of lupus encephalopathy and sustained seizures after two years. One patient developed pulmonary artery embolism four years later delivery and recovered completely.

Post-delivery, heart failure during pregnancy was recovered. Only one patient developed renal failure up to December 2013.

### 3.7. Risk Factors for Lupus Flare, Obstetric Complications and Fetal Loss in Pregnant Women with SLE (Table 6)

In the adjusted model, only SLE disease activity in the six months before pregnancy was significantly associated with lupus flare (OR 5.00, 95% CI 1.14–21.87, *p =* 0.03). New onset lupus during pregnancy was independently associated with obstetric complication (OR 7.22, 95% CI 2.14–24.38, *p =* 0.001). SLE disease activity in the six months before pregnancy was significantly associated with fetal loss (OR 5.73, 95% CI 1.42–23.05, *p =* 0.02). The association of SLE disease activity in the six months before pregnancy with fetal loss was also significant in patients without ending in therapeutic/elective abortion (OR 8.70, 95% CI 1.30–58.28, *p =* 0.03).

**Table 6:** Risk factors for lupus flare, obstetric complications and fetal loss in pregnant women with SLE.

	Unadjusted Model		Adjusted Model	
	OR (95% CI)	*p* Value	OR (95% CI)	*p* Value
***Lupus flare***				
**Age**	0.88 (0.78–0.99)	0.04	0.92 (0.80–1.06)	0.26
**Lupus nephritis**	2.44 (1.02–5.84)	0.05	1.92 (0.61–6.02)	0.26
**New onset SLE during pregnancy**	-	-	-	-
**SLE disease activity in the six months before pregnancy**	3.15 (0.80–12.42)	0.10	5.00 (1.14–21.87)	0.03
***Obstetric complications***				
**Age**	0.95 (0.83–1.07)	0.38	0.99 (0.86–1.15)	0.94
**Lupus nephritis**	2.61 (1.00–6.84)	0.05	2.31 (0.76–7.01)	0.14
**New onset SLE during pregnancy**	6.73 (2.21–20.48)	0.001	7.22 (2.14–24.38)	0.001
**SLE disease activity in the six months before pregnancy**	1.04 (0.29–3.74)	0.96	1.65 (0.39–7.09)	0.50
***Fetal loss***				
**Age**	0.86 (0.75–0.99)	0.03	0.91 (0.78–1.05)	0.18
**Lupus nephritis**	2.08 (0.83–5.17)	0.12	1.39 (0.53–3.66)	0.51
**New onset SLE duringpregnancy**	2.01 (0.69–5.84)	0.20	2.82 (0.85–9.38)	0.09
**SLE disease activity in the six months before pregnancy**	4.99 (1.44–17.30)	0.01	5.73 (1.42–23.05)	0.02
***Fetal loss* (without therapeutic/elective abortion)**				
**Age**	0.93 (0.75–1.16)	0.53	0.94 (0.74–1.20)	0.63
**Lupus nephritis**	1.12 (0.32–3.94)	0.86	0.78 (0.18–3.34)	0.73
**New onset SLE during pregnancy**	1.42 (0.25–8.03)	0.69	2.49 (0.35–17.54)	0.36
**SLE disease activity in the six months before pregnancy**	6.36 (1.16–34.81)	0.03	8.70 (1.30–58.28)	0.03

## 4. Discussion

In the current study, among women in remission for more than six months before pregnancy, 76.47% pregnancies achieved full-term deliveries and 80.39% achieved live born infants. However in women having SLE activity in the six months before pregnancy, only 23.08% pregnancies achieved full-term deliveries and 30.76% achieved live born infants. In women with new onset SLE during pregnancy, the outcome was also poor. If new onset SLE occurred during the third trimesters of pregnancy, the outcome seems better. A high rate of lupus flare during pregnancy was found in the current study. Even among women in remission for more than six months before pregnancy, the rate of lupus flare was not low (33.33%). Fortunately, psychiatric and central nervous system manifestations and *irreversible renal failure* were not found during pregnancy.

Previous studies have shown poor pregnancy outcome in women with SLE [1,2,3,4,5,7]. The pathogenesis is complex and not completely clarified. Risk factors of pregnant loss in women with SLE include active SLE, new onset SLE during pregnancy, aPLs, hypocomlementemia, anti-dsDNA antibodies, thrombocytopenia, hypertension and lupus nephritis [1,2,8,9,10,11,12]. A new study based on 992 SLE patients with 2026 pregnancies suggested that thrombocytopenia, aPL antibodies and anti-SSA antibody are associated with fetal loss in Chinese women [19]. Although women with SLE have an increased risk of adverse outcomes, patients in remission or stable mild/moderate SLE might have favorable outcomes [1,2,20]. In the current study, 80.39% pregnancies of women in remission for more than 6 months had live born infants. Apart from SLE activity, aPLs and anti-Ro/SSA antibody usually are considered highly associated with fetal loss [1,2,11]. The results of the current study also support that SLE disease activity in the six months before pregnancy is a risk factor of lupus flare and fetal loss. In the current study, some patients with SLE were followed up in other hospitals before delivery and aPSL and anti-SSA antibody were not included due to delayed date. However in group C, a high rate of anti-Ro/SSA positive was found. No neonate suffered from heart-blocker.

During pregnancy, the maternal immune system and cytokine profile are modified. These changes might account for tending to improve Th2-mediated diseases including SLE [21]. But whether or not pregnancy increases SLE relapse is still controversial. Previous studies reported varied widely rate of lupus flare during pregnancy [1,2]. Lockshin *et al*. [22,23], reported that pregnancy does not exacerbate SLE. In several prospectively studies, increased rates of lupus flare (30.8%–65%) during pregnancy were reported [3,8,24,25,26]. In some studies, the rates of lupus flare were relative lower, still 19.4%–28.3% patients suffered from lupus flare during pregnancy or post-delivery [1,2,27].

The results of the current study indicated that SLE flare is common in pregnant women with SLE. Among 62 patients (64 pregnancies) diagnosed as SLE before pregnancy, SLE flares occurred in 27 (42.19%) pregnancies. Fortunately, life threatening situation was uncommon. In patients in remission for more than six months, the prevalence of lupus flare is significantly lower. The result supports planned pregnancy after remission of SLE. The high rate of SLE flare during pregnancy might not conclude that SLE flare is induced by pregnancy because of the same high even higher rate of SLE flare observed in the control groups [28,29,30].

In the current study, pregnant women in groups B and C have a low rate of live birth. Another explanation was a higher rate of therapeutic abortions. When we excluded the pregnancies with ending in therapeutic/elective abortion, the association of SLE disease activity in the six months before pregnancy with fetal loss was still significant (OR 8.70, 95% CI 1.30–58.28, *p =* 0.03).

New onset SLE during pregnancy can be considered as SLE activity and might be associated with worse outcome [31].However, the results of the current study indicated that new onset SLE during the third trimester of pregnancy had better outcome. The other issue about new onset SLE during pregnancy is differentiate diagnose with pre-eclampsia. Lupus flare during pregnancy might have similar symptoms to pre-eclampsia. In the current study, two patients with new onset SLE during pregnancy were delayed diagnosis. SLE might be routinely ruled out in patients with pre-eclampsia.

Here, several limitations of the current study should be mentioned. First, this is only a retrospective study based on single centre. The second limitation is missing data in group A and group B. Third, there was no control group in the current study. Fourth, only a low proportion of patients were taking hydroxychloroquine during pregnancy. Fifth, we did not use serum titers of C3 and C4 to assess lupus flare during pregnancy. During pregnancy, serum titers of C3 and C4 are usually elevated, so pregnant women with lupus flare might have normal levels of C3 and C4 [15].

## 5. Conclusions

SLE should be considered a high risk of pregnancy. We also found a high frequency of lupus flare during pregnancy. If pregnancy is planned after remission for more than six months, the favorable outcome can be achieved. SLE disease activity in the six months before pregnancy was significantly associated with lupus flare and fetal loss. Unfortunately, in the current study, 20.31% (13/64) pregnancies with SLE were not adequately prepared and 23.75% (19/80) patients were diagnosed as SLE during pregnancy. New onset lupus during pregnancy was independently associated with obstetric complications.
